# Development and validation of machine learning models for predicting acute kidney injury in acute-on-chronic liver failure: a multimodel comparative study

**DOI:** 10.1080/0886022X.2025.2547262

**Published:** 2025-08-26

**Authors:** Jing Zhang, Shuxuan Tang, Jingyuan Liu, Ang Li

**Affiliations:** a Department of Critical Care Medicine, Beijing Ditan Hospital, Capital Medical University, Beijing, China; b Institute of Beijing Ditan Hospital, Capital Medical University, Beijing, China

**Keywords:** Acute kidney injury, acute-on-chronic liver failure, machine learning, prediction models, nomogram

## Abstract

**Background:**

Acute kidney injury (AKI) is one of the serious complications in acute-on-chronic liver failure (ACLF), and the mortality rate is very high. Early identification of high-risk patients is critical. Therefore, this study aimed to develop prediction models for AKI in ACLF patients based on machine learning (ML) algorithms.

**Methods:**

This retrospective study enrolled 1,076 adult patients diagnosed with ACLF, with AKI defined according to the International Club of Ascites criteria. Participants were randomly allocated into training (*n* = 753, 70%) and test (*n* = 323, 30%) sets. Six ML models were developed: logistic regression (LR), random forest (RF), k-nearest neighbors (KNN), support vector machine (SVM), decision tree (DT), and extreme gradient boosting (XGBoost). The performance of each model was assessed using the area under the receiver operating characteristic curve (AUC-ROC), the area under the precision-recall curve (AUC-PR), the calibration curve, and decision curve analysis.

**Results:**

Among participants, 250 (23.2%) developed AKI during hospitalization. Multivariate LR analyses identified ten significant variables in the training set: age, hypertension, total bilirubin, blood urea nitrogen, serum creatinine, blood uric acid, international normalized ratio, hepatic encephalopathy, abdominal infection, and sepsis. The RF model performed best in the test set (AUC-ROC = 0.899; AUC-PR = 0.806).

**Conclusions:**

The ML models can be reliable tools for predicting AKI in patients with ACLF. The RF model performed the best and can help medical clinicians to better identify patients with high risk of AKI in ACLF.

## Graphical Abstract

**Figure:**
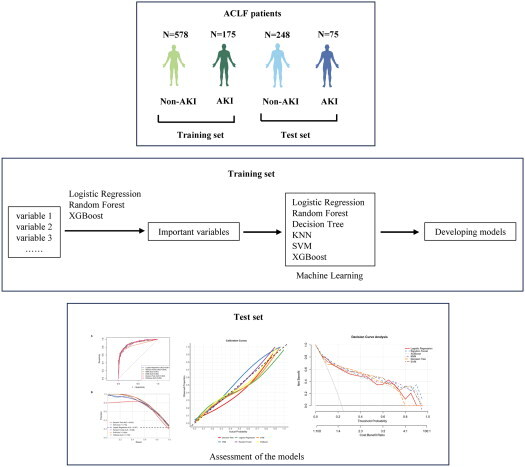

## Introduction

Acute-on-chronic liver failure (ACLF) is a clinical syndrome with acute decompensation of liver function in a short time based on chronic liver disease, often accompanied by multiple organ failure and a high mortality rate. Acute kidney injury (AKI) is one of the most common serious complications in ACLF patients, and its occurrence is related to systemic circulatory dysfunction (such as visceral artery vasodilation and increased cardiac displacement) and inflammatory response [1,2]. Studies have shown that the incidence of AKI in ACLF patients is 19.2% to 69.0%, and the mortality rate is higher than that of non-AKI patients [3–8]. Therefore, early identification and intervention of this high-risk complication among the ACLF patients is essential to improve the general prognosis.

Machine learning (ML) algorithms have demonstrated significant advantages in medical predictive modeling due to their powerful data processing and nonlinear relationship mining capabilities [9–13]. By integrating multidimensional clinical indicators, ML can extract potential risk features from massive data, providing new thinking for early warning of AKI. It is reported that the ML model has superior predictive performance in AKI compared to the traditional statistical model [14–16].

However, there is still a paucity of existing research on the development of AKI prediction models for the specific population of ACLF, and there are limited cross-sectional comparative studies on the performance of different algorithms. Therefore, in this study, we aimed to use ML algorithms to construct prediction models for AKI in patients with ACLF, provide data support for early identification of AKI in ACLF patients and individualized intervention, optimize the clinical decision-making process, and improve patients’ general prognosis.

## Methods

### Study design and participants

**Figure 1.**
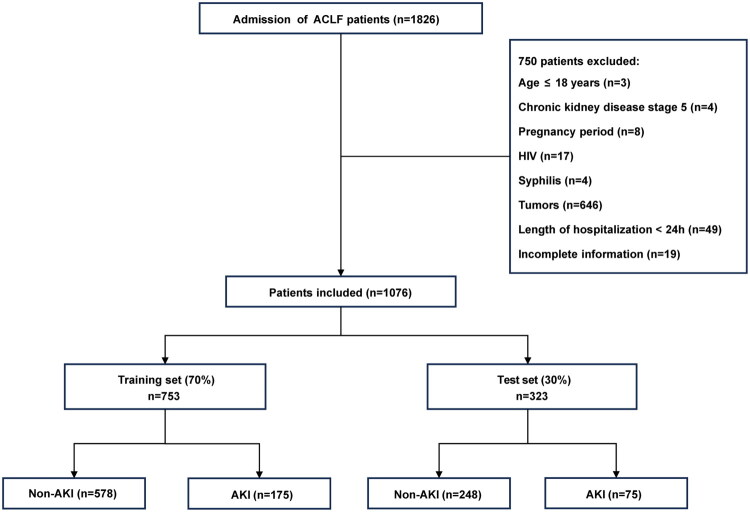
The flowchart of this study. ACLF: acute-on-chronic liver failure; AKI: acute kidney injury; HIV: human immunodeficiency virus.

This study was a retrospective analysis with data from the electronic medical record system of inpatients in Beijing Ditan Hospital affiliated with Capital Medical University, including 1,076 patients with ACLF from January 2016 to October 2024. The exclusion criteria were as follows: (1) age ≤ 18 years; (2) chronic kidney disease (CKD) stage 5; (3) pregnancy period; (4) human immunodeficiency virus (HIV); (5) syphilis; (6) tumors; (7) length of hospitalization < 24h; (8) incomplete information (Figure 1). The Ethics Committee (Beijing Ditan Hospital, Capital Medical University) approved the study at our institution (DTEC-KT2024-004-01). Written informed consent was waived for this retrospective analysis. The study was performed in accordance with the Declaration of Helsinki.

### Definitions

According to the recommendations of the Asian Pacific Association for the Study of the Liver (APASL) meeting [1], ACLF is an acute hepatic insult manifesting as jaundice (serum bilirubin ≥ 5 mg/dL (85 mmol/L)) and coagulopathy [international normalized ratio (INR) ≥ 1.5 or prothrombin activity < 40%] complicated within 4 weeks by clinical ascites and/or encephalopathy in a patient with previously diagnosed or undiagnosed chronic liver disease/cirrhosis, and is associated with a high 28-day mortality rate.

AKI was diagnosed using the International Ascites Club (ICA) definition of an increase in serum creatinine (SCr) level of ≥ 0.3 mg/dl (26.5 μmol/L) within 48 h or an increase in SCr level of more than 1.5 times the baseline value within 7 days [17]. The most recent stable SCr value within 3 months before admission can be used as the baseline SCr value. If no pre-admission creatinine value is available, the SCr value on the day of admission will be taken as the baseline value.

### Collection of the data

Clinical data of all patients were collected, including demographic data (age, gender), basic disease history (hypertension, diabetes, CKD), etiology of liver disease, cirrhosis, laboratory parameters, drug use, and complications [ascites, abdominal infection, hepatic encephalopathy (HE), gastrointestinal bleeding, sepsis, portal vein embolism].

### Statistical analysis

Variables with over 30% missing data were excluded. Variables with 10%-30% missing data were filled using multiple imputations, while those with less than 10% missing data were filled using the median. The data were randomly divided into a training set and a test set at a ratio of 7:3. The training set was used for feature selection and model construction, while the test set was used to evaluate the effectiveness of the training model. All variables were analyzed by univariate LR for the training set. Variables with *p* < 0.05 were included in subsequent covariate diagnostic analyses. Variables with a variance inflation factor (VIF) < 10 and tolerance > 0.1 were included in multivariate LR analyses to screen for independent risk variables. We uniformly used ten independent risk variables screened by multivariate LR analysis for constructing ML models and the nomogram. Six ML models were constructed, including logistic regression (LR) model, random forest (RF) model, k-nearest neighbors (KNN) model, support vector machine (SVM) model, decision tree (DT) model, and extreme gradient boosting (XGBoost) model. The models were hyper-parameter tuned by grid search and model validation was carried out by 5-fold cross-validation. The performance of the model was assessed by several metrics including the area under the receiver operating characteristic curve (AUC-ROC), the area under the precision-recall curve (AUC-PR), accuracy, sensitivity, specificity, positive predictive value (PPV), negative predictive value (NPV), and F1 score. Calibration curves were plotted to assess the predictive effectiveness of the models. Decision curve analysis (DCA) was used to analyze the clinical application of the models. We also used the SHapley Additive exPlanations (SHAP) to clarify the contributions of important variables to the model.

Normally distributed continuous variables were presented as mean ± standard deviation and compared using the independent samples t-test. Non-normally distributed continuous variables were expressed as medians with interquartile ranges [M (Q1-Q3)] and analyzed *via* the Mann-Whitney U test. Categorical variables were described as frequencies (percentages), with group comparisons performed using the χ^2^ test or Fisher exact test.

All tests were two-tailed, and a *p-*value <0.05 was considered statistically significant. All the statistical analyses were carried out with R version 4.4.2 and SPSS version 26.0.

## Results

### Baseline patient characteristics

Based on the inclusion and exclusion criteria, 1,076 patients were included in the study, and 250 patients developed AKI. The number of patients in stage 1, stage 2, and stage 3 of AKI was 98, 45, and 107, respectively. We randomized all patients into a training set (70%, 753 patients, 175 AKI, 578 non-AKI) and a test set (30%, 323 patients, 75 AKI, 248 non-AKI). Baseline characteristics of AKI and non-AKI patients in the training and test sets are shown in Table 1.

**Table 1.** Baseline characteristics of the patients with ACLF.

	Training set (*n* = 753)			Test set (*n* = 323)		
	Non-AKI (*n* = 578)	AKI (*n* = 175)	*p* value	Non-AKI (*n* = 248)	AKI (*n* = 75)	*p* value
Age (years)	49.0 (39.0–58.0)	54.0 (45.0–64.0)	<0.001	49.0 (41.0–55.0)	53.0 (45.0–65.0)	0.001
Male (n, %)	153 (26.5)	30 (17.1)	0.012	65 (26.2)	22 (29.3)	0.593
Liver Cirrhosis (n, %)	462 (79.9)	162 (92.6)	<0.001	203 (81.9)	73 (97.3)	0.001
Etiology (n, %)			<0.001			0.002
HBV	316 (54.7)	67 (38.3)	<0.001	143 (57.7)	26 (34.7)	<0.001
HCV	5 (0.9)	5 (2.9)	0.101	0 (0.0)	1 (1.3)	0.232
ALD	105 (18.2)	69 (39.4)	<0.001	53 (21.4)	23 (30.7)	0.096
AIH	28 (4.8)	3 (1.7)	0.068	13 (5.2)	4 (5.3)	1.000
PBC	15 (2.6)	4 (2.3)	1.000	1 (0.4)	3 (4.0)	0.040
Mixed	64 (11.1)	14 (8.0)	0.243	21 (8.5)	8 (10.7)	0.559
Others	45 (7.8)	13 (7.4)	0.877	17 (6.9)	10 (13.3)	0.076
CKD (n, %)	3 (0.5)	10 (5.7)	<0.001	3 (1.2)	4 (5.3)	0.090
Hypertension (n, %)	94 (16.3)	49 (28.0)	0.001	35 (14.1)	18 (24.0)	0.043
Diabetics (n, %)	80 (13.8)	38 (21.7)	0.012	29 (11.7)	8 (10.7)	0.807
NSAIDs (n, %)	32 (5.5)	8 (4.6)	0.618	16 (6.5)	8 (10.7)	0.223
Anti-hepatitis virus drugs (n, %)	340 (58.8)	59 (33.7)	<0.001	148 (59.7)	28 (37.3)	0.001
Diuretic (n, %)	485 (83.9)	157 (89.7)	0.058	216 (87.1)	68 (90.7)	0.406
ACEI/ARB (n, %)	18 (3.1)	5 (2.9)	0.863	13 (5.2)	3 (4.0)	0.664
WBC count (× 10^9^/L)	6.1 (4.5–8.4)	7.9 (5.8–12.0)	<0.001	6.1 (4.4–8.1)	8.9 (5.7–13.8)	<0.001
HB (g/L)	122.0 (100.4–140.0)	100.0 (77.4–130.0)	<0.001	120.4 (103.3–140.0)	96.4 (80.0–119.6)	<0.001
PLT count (× 10^9^/L)	101.5 (66.3–152.0)	89.0 (47.4–142.0)	0.015	99.0 (67.0–144.8)	78.0 (52.0–123.0)	0.015
TBIL (μmol/L)	235.6 (174.2–331.3)	290.1 (215.4–403.8)	<0.001	248.7 (193.4–350.6)	248.0 (184.3–413.8)	0.222
ALT (U/L)	137.1 (47.3–526.7)	61.5 (32.3–242.3)	<0.001	149.2 (44.6–551.8)	52.6 (24.0–114.2)	<0.001
AST (U/L)	159.9 (77.6–440.2)	108.2 (59.3–346.3)	0.002	175.6 (86.7–454.6)	100.3 (53.0–182.0)	<0.001
Albumin (g/L)	30.9 (27.5–33.9)	29.2 (25.6–32.4)	<0.001	30.3 (27.3–33.1)	28.3 (24.2–31.3)	<0.001
CRP (mg/L)	12.4 (7.1–22.9)	24.2 (11.7–47.0)	<0.001	12.7 (7.2–21.6)	23.2 (12.9–48.7)	<0.001
Baseline BUN (mmol/L)	4.3 (3.1–5.9)	12.0 (6.2–17.9)	<0.001	4.3 (3.3–5.9)	10.9 (6.3–17.8)	<0.001
Baseline SCr (μmol/L)	65.4 (54.5–76.8)	85.8 (72.4–127.1)	<0.001	66.6 (56.4–79.1)	96.5 (72.9–155.1)	<0.001
Baseline eGFR (mL/min/1.73 m²)	105.9 (94.0–117.0)	84.5 (52.3–103.0)	<0.001	107.0 (93.0–117.9)	77.0 (40.9–94.0)	<0.001
Blood uric acid (μmol/L)	163.0 (113.0–233.3)	315.0 (178.0–507.0)	<0.001	154.5 (109.0–236.8)	301.0 (179.0–448.0)	<0.001
INR	2.2 (1.9–2.7)	2.4 (1.9–3.1)	0.006	2.2 (1.8–2.7)	2.1 (1.8–2.7)	0.485
Complications (n, %)						
Ascites	522 (90.3)	169 (96.6)	0.008	206 (83.1)	73 (97.3)	0.002
HE	195 (33.7)	131 (74.9)	<0.001	82 (33.1)	51 (68.0)	<0.001
Gastrointestinal bleeding	47 (8.1)	76 (43.4)	<0.001	15 (6.0)	32 (42.7)	<0.001
Abdominal infection	321 (55.5)	158 (90.3)	<0.001	130 (52.4)	67 (89.3)	<0.001
Sepsis	13 (2.2)	51 (29.1)	<0.001	1 (0.4)	19 (25.3)	<0.001
Portal vein embolism	12 (2.1)	8 (4.6)	0.126	7 (2.8)	3 (4.0)	0.892

AKI: acute kidney injury; HBV: hepatitis B virus; HCV: hepatitis C virus; ALD: alcoholic liver disease; AIH: autoimmune hepatitis; PBC: primary biliary cirrhosis; CKD: chronic kidney disease; NSAIDs: nonsteroidal antiinflammatory drugs; ACEI: angiotensin converting enzyme inhibitors; ARB: angiotensin II receptor blockers; WBC: white blood cell; HB: hemoglobin; PLT: platelet; TBIL: total bilirubin; ALT: alanine amino transaminase; AST: aspartate transaminase; CRP: C-reactive protein; BUN: blood urea nitrogen; SCr: serum creatinine; eGFR: estimated glomerular filtration rate; INR: international normalized ratio; HE: hepatic encephalopathy.

The age of non-AKI and AKI patients in the training set and test set was 49.0 (39.0–58.0) years, 54.0 (45.0–64.0) years, 49.0 (41.0–55.0) years and 53.0 (45.0–65.0) years, respectively. Among the ACLF patients, those with AKI were older, had a higher percentage of combined cirrhosis, hypertension, ascites, HE, gastrointestinal bleeding, abdominal infection, and sepsis, a lower percentage of hepatitis B virus (HBV) infection and the use of anti-hepatitis virus drugs, higher levels of white blood cell (WBC) count, C-reactive protein (CRP), blood urea nitrogen (BUN), SCr, and blood uric acid, and lower levels of hemoglobin, platelet (PLT) count, alanine amino transaminase (ALT), aspartate transaminase (AST), albumin, and estimated glomerular filtration rate (eGFR).

### Establishment of clinical predictor variables

Univariate logistic regression analysis of the training set showed that the following 22 variables were significantly associated with AKI in ACLF patients (*p* < 0.05): age (*p* < 0.001), male (*p* = 0.012), cirrhosis (*p* < 0.001), etiology (*p* < 0.001), CKD (*p* < 0.001), hypertension (*p* = 0.001), diabetics (*p* = 0.013), WBC (*p* < 0.001), HB (*p* < 0.001), TBIL (total bilirubin) (*p* < 0.001), albumin (*p* < 0.001), CRP (*p* < 0.001), baseline BUN (*p* < 0.001), baseline SCr (*p* < 0.001), baseline eGFR (*p* < 0.001), blood uric acid (*p* < 0.001), INR (*p* = 0.001), ascites (*p* = 0.012), HE (*p* < 0.001), gastrointestinal bleeding (*p* < 0.001), abdominal infection(*p* < 0.001), sepsis(*p* < 0.001).

Those variables with *p* < 0.05 in the univariate logistic analysis were included in the multivariate logistic regression analysis, and the results showed that age [odds ratio (OR), 1.029; 95% confidence interval (CI), 1.007–1.051; *p* = 0.010], hypertension (OR, 1.976; 95% CI, 1.093–3.572; *p* = 0.024), TBIL (OR, 1.008; 95% CI, 1.005–1.010; *p* < 0.001), baseline BUN (OR, 1.180; 95% CI, 1.106–1.259; *p* < 0.001), baseline SCr (OR, 1.011; 95% CI, 1.001–1.022; *p* = 0.030), blood uric acid (OR, 1.006; 95% CI, 1.004–1.008; *p* < 0.001), INR (OR, 1.339; 95% CI, 1.071–1.673; *p* = 0.010), HE (OR, 2.683; 95% CI, 1.539–4.676; *p* < 0.001), abdominal infection (OR, 3.189; 95% CI, 1.514–6.717; *p* = 0.002), and sepsis (OR, 9.646; 95% CI, 4.001–23.259; *p* < 0.001) were independently associated with AKI development in ACLF patients (Table 2).

**Table 2.** Multivariate logistic regression analysis on the risk variables for the occurrence of AKI in ACLF patients.

Variables	OR	95% CI for OR		*p* value
		Lower limit	Upper limit	
Age	1.029	1.007	1.051	0.010
Baseline BUN	1.180	1.106	1.259	<0.001
Baseline SCr	1.011	1.001	1.022	0.030
Blood uric acid	1.006	1.004	1.008	<0.001
INR	1.339	1.071	1.673	0.010
TBIL	1.008	1.005	1.010	<0.001
Hypertension	1.976	1.093	3.572	0.024
HE	2.683	1.539	4.676	<0.001
Abdominal infection	3.189	1.514	6.717	0.002
Sepsis	9.646	4.001	23.259	<0.001

OR: odds ratio; CI: confidence interval.

**Figure 2.**
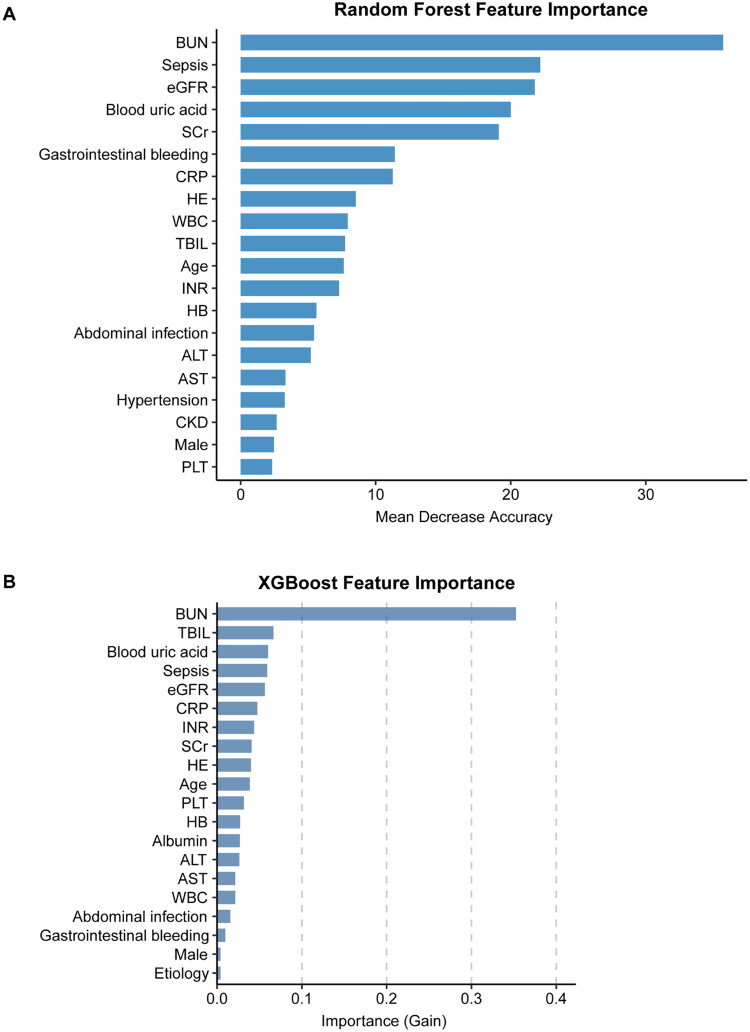
(A) The feature importance derived from the Random Forest model. (B) The feature importance derived from the XGBoost model. BUN: blood urea nitrogen; eGFR: estimated glomerular filtration rate; SCr: serum creatinine; CRP: C-reactive protein; HE: hepatic encephalopathy; WBC: white blood cell; TBIL: total bilirubin; INR: international normalized ratio; HB: hemoglobin; ALT: alanine amino transaminase; AST: aspartate transaminase; CKD: chronic kidney disease; PLT: platelet; XGBoost: extreme gradient boosting.

Additionally, this study also listed the top 20 important variables of the RF and XGBoost models (Figure 2). BUN, sepsis, eGFR, blood uric acid, SCr, CRP, HE, TBIL, age, and INR were significant predictors of RF and XGBoost algorithms.

### Model performance

**Figure 3.**
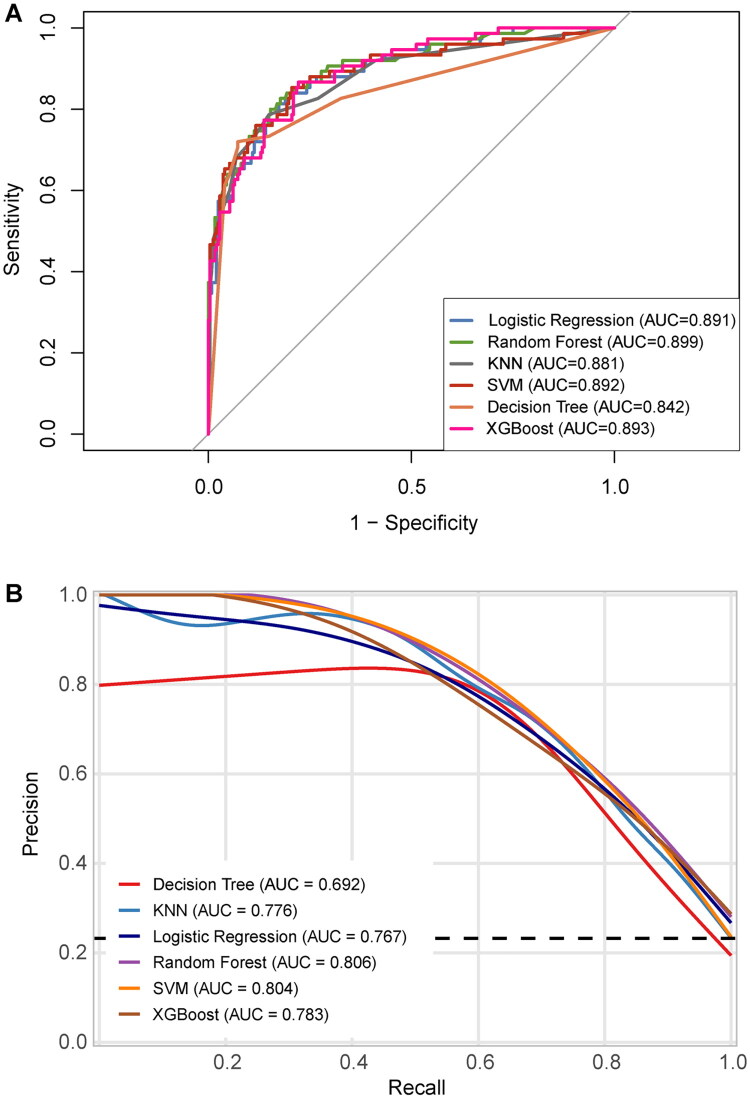
(A) Receiver operating characteristic curves of the six models in the test set. (B) The precision-recall curves of the six models in the test set. KNN: k-nearest neighbors; SVM: support vector machine; XGBoost: extreme gradient boosting; AUC: area under the curve.

Table 3 displayed the 6 ML models’ predictive performance. The AUC-ROC values of the LR, RF, KNN, SVM, DT, and XGBoost models were 0.891, 0.899, 0.881, 0.892, 0.842, and 0.893, respectively (Figure 3A). For the AUC-PR, the corresponding values for the same models were 0.767, 0.806, 0.776, 0.804, 0.692, and 0.783 (Figure 3B). Among the 6 ML models, the RF model achieved the highest performance, with an accuracy of 0.824, sensitivity of 0.827, specificity of 0.823, PPV of 0.585, NPV of 0.940, and an F1 score of 0.685.

**Table 3.** Model performance metrics.

Models	AUC-ROC	AUC-PR	Accuracy	Sensitivity	Specificity	PPV	NPV	F1 score
Logistic regression	0.891	0.767	0.824	0.813	0.827	0.587	0.936	0.682
Random forest	0.899	0.806	0.824	0.827	0.823	0.585	0.940	0.685
KNN	0.881	0.776	0.836	0.787	0.851	0.615	0.930	0.690
SVM	0.892	0.804	0.808	0.853	0.794	0.557	0.947	0.674
Decision tree	0.842	0.692	0.879	0.720	0.927	0.750	0.916	0.735
XGBoost	0.853	0.783	0.793	0.853	0.774	0.533	0.946	0.656

KNN: k-nearest neighbors; SVM: support vector machine; XGBoost: extreme gradient boosting; AUC: area under the curve; ROC: receiver operating characteristic; PR: precision-recall; PPV: positive predictive value; NPV: negative predictive value.

### Development of the nomogram and clinical application of multiple ML models

**Figure 4.**
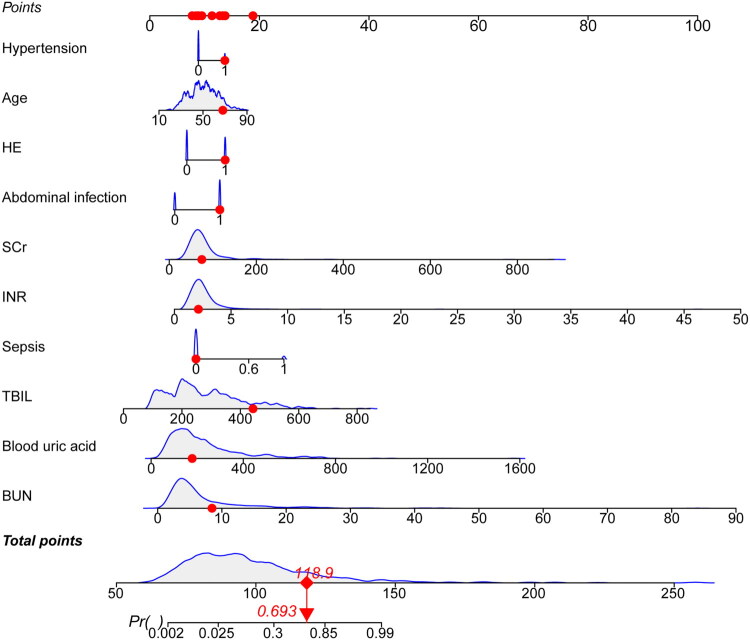
Nomogram for the early prediction of AKI in patients with ACLF.

The nomogram of ACLF-AKI was drawn using the ten variables obtained by screening, as shown in Figure 4. In the nomogram, an option for each variable corresponds to a corresponding score, and the total score is obtained by adding the scores for all ten variables. At the bottom of the nomogram, probability predictions corresponding to different total scores are given. The higher the total score, the higher the risk of AKI in ACLF patients. For example, a 68-year-old patient with ACLF has a TBIL level of 443.4 μmol/L, a BUN level of 8.5 mmol/L, a SCr level of 74.9 μmol/L, a blood uric acid level of 178 μmol/L, and an INR level of 2.1 in the presence of hypertension, HE, and abdominal infection without sepsis. The corresponding points for this patient are then 13.6, 19.2, 11.2, 9.6, 7.6, 8.7, 14.0, 14.0, 13.0, and 8.0, for a total point of 118.9. This means that the estimated probability of AKI occurring in this patient is approximately 69.3%.

**Figure 5.**
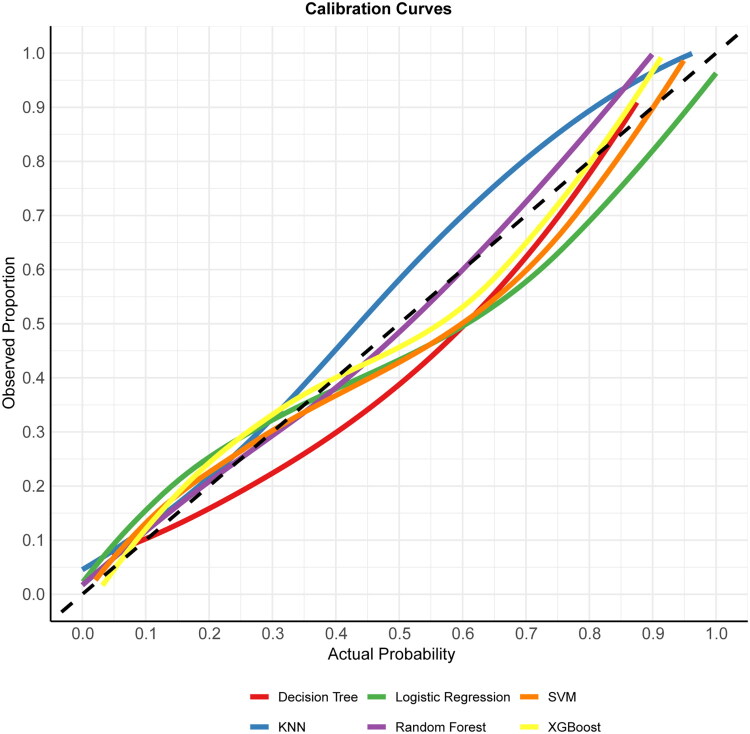
Calibration curves of the models in the test set. The dashed line represents full calibration. Each colored line represents the calibration curve for a particular model. The closer the colored line is to the dashed line, the better the calibration performance of the model.

**Figure 6.**
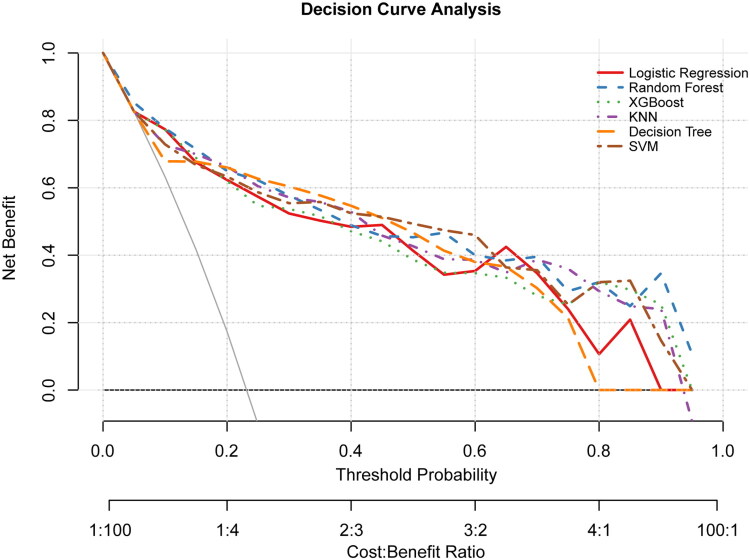
Decision curve analyses of the models in the test set. The X-axis represents the threshold probability, and the Y-axis represents the net benefit. The solid gray line represents the net benefit of treating all patients, and the dashed gray line represents the net benefit of not treating any patients. Each colored line represents the net benefit of a particular model at different threshold probabilities. The model with the highest net benefit for a given threshold probability is considered the optimal model for that probability.

The calibration curves of LR model (Brier score = 0.1020), RF model (Brier score = 0.0938), KNN model (Brier score = 0.0993), SVM model (Brier score = 0.0944), DT model (Brier score = 0.1007), and XGBoost model (Brier score = 0.1002) in the test set are shown in Figure 5. A smaller value of the Brier score represents better calibration. Compared with other models, the actual prediction curve of the RF model was more consistent with the ideal curve, indicating that the RF prediction model had good accuracy. According to the DCA curves (Figure 6), the RF and XGBoost models have significantly higher net gains than the other models in the intermediate threshold probability range (approximately 0.1 to 0.7), suggesting that using these two models for decision making in this interval leads to greater net clinical gains. The LR model also performed better in the intermediate threshold interval, but slightly lower than the RF and XGBoost models. The KNN, DT, and SVM models performed poorly and may even have been below the two reference lines in some threshold ranges.

### SHAP values evaluate feature importance

**Figure 7.**
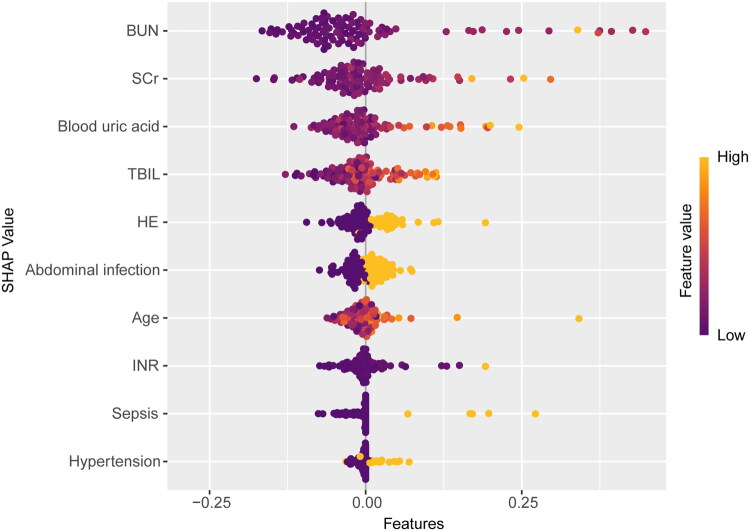
The SHAP plot of the Random Forest model in the training set. SHAP: SHapley Additive exPlanations.

As shown in Figure 7, the SHAP plot of the RF model showed the distribution of SHAP values for each feature to help understand the contribution of each feature to the model predictions. The orange dots represented high eigenvalues, while the dark purple dots represented low eigenvalues. The ranking of feature importance showed that BUN, SCr, and blood uric acid were the top three important features, followed by TBIL, HE, abdominal infection, age, INR, sepsis, and hypertension.

## Discussion

In this study, six ML models were developed to predict the risk of AKI in ACLF patients. The results showed that the RF model had the best predictive performance (AUC-ROC = 0.899) in the test set. The calibration curves and DCA curves also suggested that the RF model had a higher predictive value. Current literature reveals limited applications of ML algorithms for ACLF-AKI prediction. To the best of our knowledge, this represents the first comprehensive comparison of multiple ML methods for developing AKI prediction models in ACLF, which potentially provides more precise timing for the early identification of AKI.

AKI is a common and serious clinical event associated with the survival rate of ACLF patients. Using diagnostic criteria established by the APASL for ACLF and the ICA criteria for AKI, our analysis identified that the incidence of AKI during hospitalization in patients with ACLF was 23.2%. According to previous studies, the incidence of AKI in ACLF patients ranges from 19.2% to 69.0% [3–8]. Currently, there are varying definitions of ACLF because there is no universally accepted diagnostic criteria for it [18]. The definition of AKI is also continuously updated. In 2004, the Acute Kidney Injury Network (AKIN) expert group proposed the AKIN criteria for diagnosing AKI [19]. In 2012, the Kidney Disease Improving Global Prognosis Organization (KDIGO) proposed new diagnostic criteria for AKI based on the AKIN criteria, and the KDIGO criteria [20]. Since that time, the ICA has revised the diagnostic criteria for AKI in patients with cirrhosis several times, eventually releasing the ICA-AKI diagnostic criteria in 2015 [17], which improved the diagnostic rate of AKI. The discrepancy in the incidence of AKI in patients with ACLF may be due to the different definitions of ACLF and AKI used in different research groups and institutes.

In the area of AKI screening among ACLF patients, we found ten variables which were strongly associated with AKI. TBIL, a key biomarker of cholestasis, demonstrates renal pathological correlations including bile cast formation and tubular epithelial damage [21,22]. And the study found that cholestasis is associated with dysfunction of proximal renal tubule cells, which can cause Fanconi syndrome [23]. INR is a well-known marker of progressive liver failure and therefore worsening portal hypertension which has been linked to renal impairment [24,25]. Elevated BUN and SCr usually indicate decreased glomerular filtration rate and impaired renal function, with SCr elevation severity directly corresponding to AKI staging and prognostic outcomes [26,27]. However, the specificity of BUN and SCr is limited because changes in BUN and creatinine levels are influenced by many factors, including age, gender, race, muscle activity, medications, blood volume, nutritional status, and protein intake.

Systemic infections, particularly sepsis and spontaneous bacterial peritonitis, emerge as critical AKI precipitants in ACLF through multifactorial mechanisms including microcirculatory disturbance, mitochondrial dysfunction, and cytokine-mediated inflammation [28–30]. Our analysis identified HE as an independent AKI risk factor, which is consistent with the findings in patients with cirrhosis [31]. In terms of mechanism, studies have found that ammonia can upregulate arginase-2 in human proximal tubular epithelial cells while inducing a decrease in tubular injury markers and tubular function markers [32]. Additionally, ammonia, along with various other potential factors (inflammation, oxidative/nitrative stress, bile acids), may cause arginine deficiency and decreased endothelial nitric oxide synthase activity, promoting microcirculatory dysfunction/injury, tissue hypoxia, and tubular injury accompanied by inflammation. Hyperuricemia not only reflects abnormal purine metabolism but also exacerbates local inflammatory responses by activating the NOD-like receptor thermal protein domain associated protein 3 (NLRP3) inflammasome within the kidney through crystal deposition [33]. It has also been reported that uric acid can further cause renal injury by promoting the expression of interleukin-17 [34]. Hypertension is the second leading cause of end-stage renal disease (ESRD) after diabetes [35]. Activation of the renal angiotensin/aldosterone system by angiotensin II/angiotensin type 1 receptor signaling, the aldosterone/mineralocorticoid receptor pathway, and a positive relationship between oxidative stress and inflammation are the major interacting pathophysiological mechanisms of renal injury in hypertension [36,37]. The positive association between aging and AKI risk may be due to the additive effect of decreased organ reserve function and underlying diseases (such as diabetes and arteriosclerosis) in elderly patients [38,39].

These risk factors interact and influence each other, and jointly promote the occurrence and development of AKI. The further constructed nomogram may be a simple and reliable tool to identify AKI in ACLF patients. The AKI prediction models for patients with ACLF can serve as a foundation for making informed decisions about optimizing individualized surveillance programs for individuals at risk for AKI. By facilitating early identification of high-risk populations, clinicians can customize treatment plans based on the models’ risk stratification. For high-risk patients, clinicians can adopt targeted intervention strategies, including but not limited to: initiating early nephrology specialty consultations, implementing individualized fluid management protocols, strictly avoiding or cautiously using nephrotoxic drugs and enhancing protective measures (e.g. adequate hydration) when they are needed, and increasing the frequency of monitoring patients’ urine output and renal function indices, among other management measures [40]. For low-risk patients, routine monitoring may be sufficient. This approach can help avoid unnecessary medical interventions and resource utilization, while also reducing patient burden.

This study has several strengths. First, we pioneered the integration of ML algorithms with multidimensional clinical parameters to construct predictive models for AKI in ACLF patients. These models can improve clinical decision-making by stratifying AKI risk using routine clinical data. Furthermore, we developed an interpretable nomogram that visually represents prediction outcomes, allowing clinicians to precisely quantify individual AKI risks and optimize personalized therapeutic strategies.

However, this study also has limitations. Firstly, this study may have suffered from selection bias due to its retrospective single-center design and limited the validation efficacy of subgroups (e.g., sepsis- or HBV-related subgroups) (due to its potentially insufficient sample size). Therefore, further validation will be performed by multicenter, prospective cohort studies in the future. Secondly, despite robust model performance, the models were trained on a dataset with an imbalanced AKI prevalence of 23.2%. Due to concerns regarding the generation of synthetic data in clinical prediction contexts, more advanced techniques like SMOTE were not used, potentially reducing sensitivity for detecting AKI cases [41]. While we conducted internal validation of six ML models, the lack of external validation restricted the models’ clinical applicability. Thirdly, because data from different periods were not utilized for time validation, this study could not assess the model’s stability as it evolved in clinical practice. As a result, it is challenging to guarantee the reliability of its long-term predictive utility. Fourthly, variable screening in this study relied on traditional LR; however, penalized regression methods, such as least absolute shrinkage and selection operator (LASSO) or ridge regression, were not explored. These methods may help reduce the risk of overfitting by shrinking the coefficients. Future studies should compare the effectiveness of various modeling strategies in multicenter cohorts. Finally, although SCr remains the diagnostic criterion for AKI classification, future studies should incorporate emerging biomarkers and clinical features to improve early detection accuracy.

## Conclusion

In this study, we developed prediction models of AKI for ACLF patients using ML algorithms. The RF model demonstrated the best predictive performance, which could assist clinicians in identifying AKI patients more precisely at the early stage. This study provides a validated, interpretable, and clinically relevant framework that can inform future development of predictive decision-support tools in hepatology and critical care.

## Supplementary Material

Supplementary material.xlsx

## Data Availability

The study data may be provided by contacting the corresponding author upon reasonable request.
